# Peer-mentoring Program during the Preclinical Years of Medical School at Bonn University: a Project Description

**DOI:** 10.3205/zma001154

**Published:** 2018-02-15

**Authors:** Hendrik Lapp, Philipp Makowka, Florian Recker

**Affiliations:** 1 Helios Clinic Krefeld, Department for internal medicine I, Cardiology, Krefeld, Germany; 2 University Hospital Frankfurt, Department for internal medicine II, Haematology and Oncology, Frankfurt on the Main, Germany; 3 University Hospital Bonn, Department for Gynaecology and Obstetrics, Bonn, Germany

**Keywords:** Peer teaching, mentoring, preclinical semesters, educational cases, medical school

## Abstract

**Introduction: **To better prepare young medical students in a thorough and competent manner for the ever increasing clinical, scientific, as well as psychosocial requirements, universities should enable a close, personal transfer of experience and knowledge. Structured mentoring programs are a promising approach to incorporate clinical subjects earlier into the preclinical training. Such a mentoring program facilitates the prioritization of concepts from a broad, theory-heavy syllabus.

Here we report the experiences and results of the preclinical mentoring program of Bonn University, which was introduced in the winter semester of 2012/2013.

**Project desciption: **The program is characterized by the concept of peer-to-peer teaching during the preclinical semesters of medical school. Regular, voluntary course meetings with different clinical case examples provide students the opportunity to apply knowledge acquired from the basic science curricula; furthermore, a personal contact for advice and support is ensured. Thus, an informal exchange of experiences is made possible, which provides to the students motivational and learning aids, in particular for the oral examination at the end of the premedical semesters as well as for other examinations during medical school.

**Results: **Over the course of the preceding three years the number of participants and the interest in the program grew steadily. The analysis of collected evaluations confirms very good communication between mentors and students (>80%), as well as consistently good to very good quality and usefulness in terms of the mentors’ subject-specific and other advice. The overall final evaluation of the mentoring program was always good to very good (winter semester: very good 64.8±5.0%, good 35.2±5.0%, summer semester: very good 83.9±7.5%, good 16.1±7.5%)

**Summary:** In summary, it has been shown that the mentoring program had a positive impact on the development, education and satisfaction of students beginning their preclinical semesters at Bonn University.

## Zusammenfassung

**Einleitung: **Um junge Medizinstudierende auf die stetig wachsenden Anforderungen eines Arztes klinisch, wissenschaftlich sowie psycho-sozial allumfassend und kompetent besser vorzubereiten, sollten Universitäten eine enge, persönliche Erfahrungs- und Wissensvermittlung ermöglichen. Strukturierte Mentorenprogramme als Lösungsmodell klinische Aufgabenfelder früher in die vorklinische Lehre einfließen zu lassen, um somit eine begleitete Priorisierung des breiten, theoretisch geprägten universitären Lehrstoffes zu erleichtern, stellen einen vielversprechenden Ansatzpunkt dar.

Hier berichten wir über die Erfahrungen und Ergebnisse des vorklinischen Mentorenprogrammes der Universität Bonn, welches zum Wintersemester 12/13 eingeführt wurde.

**Projektbeschreibung: **Das Programm zeichnet sich durch das Konzept des peer-to-peer-Teachings in den Semestern der Vorklinik im Rahmen eines humanmedizinischen Regelstudienganges aus. In regelmäßigen, freiwilligen Kurstreffen mit verschiedenen klinischen Fallbeispielen soll Studierenden die Möglichkeit geboten werden, erworbene Kenntnisse aus den curricularen Grundlagenfächern eigenständig anzuwenden, sowie einen Kontakt mit einem persönlichen Ansprechpartner für Ratschläge und Hilfestellung zu gewährleisten. Auf diese Weise wird ein ungezwungener Erfahrungsaustausch ermöglicht, der den Studierenden eine Motivations- und Lernhilfe bietet, insbesondere für die mündliche Physikumsprüfung sowie für weitere Prüfungen des Studiums.

**Ergebnisse: **Über die letzten drei Jahre konnte die Teilnehmerzahl und das Interesse am Programm stetig gesteigert werden. Die Auswertung der gesammelten Evaluationen bestätigt eine sehr gute Kommunikation zwischen Tutor und Studierenden (über 80%), sowie durchweg gute bis sehr gute Qualität und Nützlichkeit der fachlichen, als auch sonstigen Tipps der Mentoren. Eine abschließende Bewertung der Erwartungen an das Mentorenprogramm wurde insgesamt auf einer Schulnotenskala stets als gut bis sehr gut bewertet (Wintersemester: sehr gut 64.8±5.0%, gut 35.2±5.0%, Sommersemester: sehr gut 83.9±7.5%, gut 16.1±7.5%)

**Zusammenfassung:** Zusammenfassend hat sich gezeigt, dass sich das Mentorenprogramm positiv auf die Entwicklung, Ausbildung und Zufriedenheit der Studienanfänger in der Bonner Vorklinik auswirkt.

## Introduction

Medical students are confronted with ever increasing challenges along their career paths secondary to expanding competency requirements. This increased work and academic burden means that medical students need additional support [1], [2]. Particularly during the basic sciences years, support and mentoring are seldom available. In some countries, the age of medical students is becoming younger, and the students may also lack some of the self-reliance skills that come with maturity [3], [4], [5]. The World Federation for Medical Education recommends structured and formal mentoring programs during medical education [6]. Furthermore, they can serve as good career catalysts [7], as close contacts are developed early and continue over the semesters. Stressors encountered early in medical school, such as starting a new life in an unknown town or performing literature searches are alleviated by structured, peer-to-peer mentoring programs [8], [9]. The employed mentors serve as a fundamental contact persons and counsellors for younger fellow students [10]. They work in coordination with academic staff to prioritize subject matter for students.

To further improve the integrated teaching of natural sciences and medicine at Bonn University, a peer-to-peer led mentoring program was introduced in cooperation with curricular management under the sponsorship of the Institute for Physiology II. This optional program was continuously refined during the last three years and successfully offered as part of the preclinical years at Bonn University. Here we present the results of this preclinical mentoring program and discuss them in the context of preclinical education. 

## Description of the project

This preclinical mentoring program at Bonn University medical school was introduced as optional meetings starting in the 2012/2013 winter semester. It has continued without interruption and was successively expanded with new offerings and subjects. It entails regular course meetings with first and second semester students, as well as an all-encompassing real-time simulation of the oral preclinical sciences exam for fourth semester students. Ten students in the more advanced clinical semesters independently develop, organize, and direct the offered classes and simulations, which are subsequently evaluated in group meetings. The mentors are contact persons for the younger students, provide counsel, help them to settle into university life in an unknown city, and work with them to develop individualized structures for learning and organization. For this work, the mentors are officially employed as student assistants at the Institute of Physiology II and are paid for by grants from a “Qualitätspakt Lehre” (quality – pact teaching) program of the federal state of North Rhine –Westphalia. 

To ensure contact, students are invited to six seminars and one practical day (Praxistag) distributed across the semester. Participants sign up at the beginning of the semester according to their class schedule with the mentor of their choice. Lists with the seminar information (date, time, mentor) are made available in the student council office for one week. Students sign up by entering their name and email address. The size of the resulting groups are 5-20 participants. Groups that are too large are divided, groups that are too small are combined. The aim is to provide the students plenty of opportunities distributed over the course of the semester for group conversations or private conversations with the mentors. Opportunities for these conversations are provided immediately after the seminar meetings. The seminars deal with a clinical question using an example case. The students apply the knowledge they obtained from the basic sciences to solve the question with the support of the mentor. A central element of the seminar is for the students to be motivated to ask questions and to apply basic science knowledge so as to develop their own approaches to solve problems [11]. Thus, the students are shown that the preclinical curriculum is an important basis for many later tasks as a physician. The courses are designed to arouse the students‘ interest and curiosity and to facilitate an exchange of experiences during the seminars. The mentors can thus decrease reserve and provide subjects for discussion. The resulting discussions regarding orientation at the university can be continued in an informal setting after the seminars. 

A prepared PowerPoint presentation supports the seminar; however, it only serves as a guide for the subject. Every mentor is free to organize his seminar individually and to incorporate information from his own medical school experience. Thus, the mentor’s clinical and research experience is integrated into the subject of the seminar. Mentors do not receive specific didactic instruction, but rather they are to pass on the imprint of their prior training. However, mentors meet regularly to review the students‘ evaluations of the basic seminar contents, organization, and the subjects discussed. If appropriate, adjustments are made.

At the beginning of the seminar, the case example is illustrated either by video, by the mentor acting, or by a detailed written description. Subsequently, the students practice gathering information regarding the patient’s history and form a rough differential diagnosis (infection, trauma, genetic cause, etc.). Thus, the students have the opportunity to demonstrate orally their basic science knowledge in order to develop a differential diagnosis and to improve it with the mentor’s help. The cases are completed when the subject has been conclusively discussed and there are no further questions by the participants. Currently, the average duration of a seminar is about two hours.

The seminars in the first and second semesters deal with the subjects listet in Table 1 (Tab. 1) (the related preclinical sciences are shown in italics) - status as of summer semester 2016.

Every semester students are also invited to participate in a practice day to apply their theoretical knowledge. The practice day during the first semester deals with taking a structured patient history followed by the necessary, discipline-specific physical examination with a focus on the most important organs, e.g. cardiac auscultation, abdominal palpation, orthopedic joint examination as well as the basic neurologic examination including reflex status. During the second semester students apply Basic Life Support techniques on a patient simulator at the Skills Lab of Bonn University. In addition, simple everyday accidents/incidents (e.g. insect bite, sprained foot, etc.) and their acute and correct treatments are discussed. It should be pointed out that during the course of the program development courses with the same basic concept but different cases were extended to dental students. Due to the limited number of semesters no further description or evaluation is provided here.

In addition, a comprehensive real-time simulation of the oral examination at the end of the preclinical years is offered to students in the fourth semester. This is conducted in real-time according to the specifications of the state’s examination office. Anatomy, physiology and biochemistry are tested. Besides aiding reflection on the state of one’s knowledge, the simulation is also supposed to improve verbalization of complex issues in a structured manner, to present approaches for dealing with questions that cannot be answered directly, and to help deal with the psychological pressure of a state examination. To that end, after the simulation there is a one hour meeting for feedback between the participants and mentors. A special focus is placed on the presentation of the participant including gestures and behavior during the examination, as well as basics such as medical terminology or the labeling of axes on graphs.

In order to help students to become more articulate for oral examinations, the “EMMA” seminars (“**E**infach **m**al den **M**und **a**ufmachen”, “Just open your mouth”) were introduced starting in the summer semester 2016. This was based on students’ wishes as expressed in the evaluations in the preceding semesters. These voluntary seminars are offered during the fourth preclinical semester; in addition to seminars and interdisciplinary and small group practice sessions that are already part of the medical school curriculum. Due to their novelty, the EMMA-seminars will not be further discussed here.

In summary, the preclinical mentoring program at Bonn University serves as a platform to motivate students to see the basic sciences of the medical school curriculum in a more interesting and diverse way, to practice clinical soft skills such as structured history taking or conversational technique in patient management, as well as to support specifically preparation for the oral part of the first state examination.

## Results

From the start, a structured evaluation was conducted in cooperation with the Center for Evaluation and Methods (Zentrum für Evaluation und Methoden (ZEM)) of Bonn University. This was done to guarantee quality assurance, to incorporate the wishes and suggestions submitted by the participants, and to guarantee the comparability with other educational events. The evaluation was done in a pencil-to-paper format immediately after the end of every seminar and was collected by the mentors (response rate 100%). At the end of the semester, a summary analysis was performed by the ZEM of all 10 seminar groups. Answers on the questionnaires could be provided as ++ (very good), + (good), - (bad), -- (very bad) as well as “I do not know”. The results without abstentions (“I do not know”) are presented as summary data for the last three years. Due to different numbers of participants during the semesters, the results are given as mean percentages ± standard error of the mean for the winter semesters (WS) 2012/13, 2013/14 and 2014/15, and for the summer semesters (SS) 2013, 2014 und 2015. The questionnaires were filled out and analyzed anonymously. Therefore, no analysis of sociographic data is possible, and the presented results are a cross-section of all participants. Statistical analysis was done using the unpaired, two-tailed t-test with Welch correction. A p<0.05 was considered to indicate statistical signifcance.

**Figure 1:**
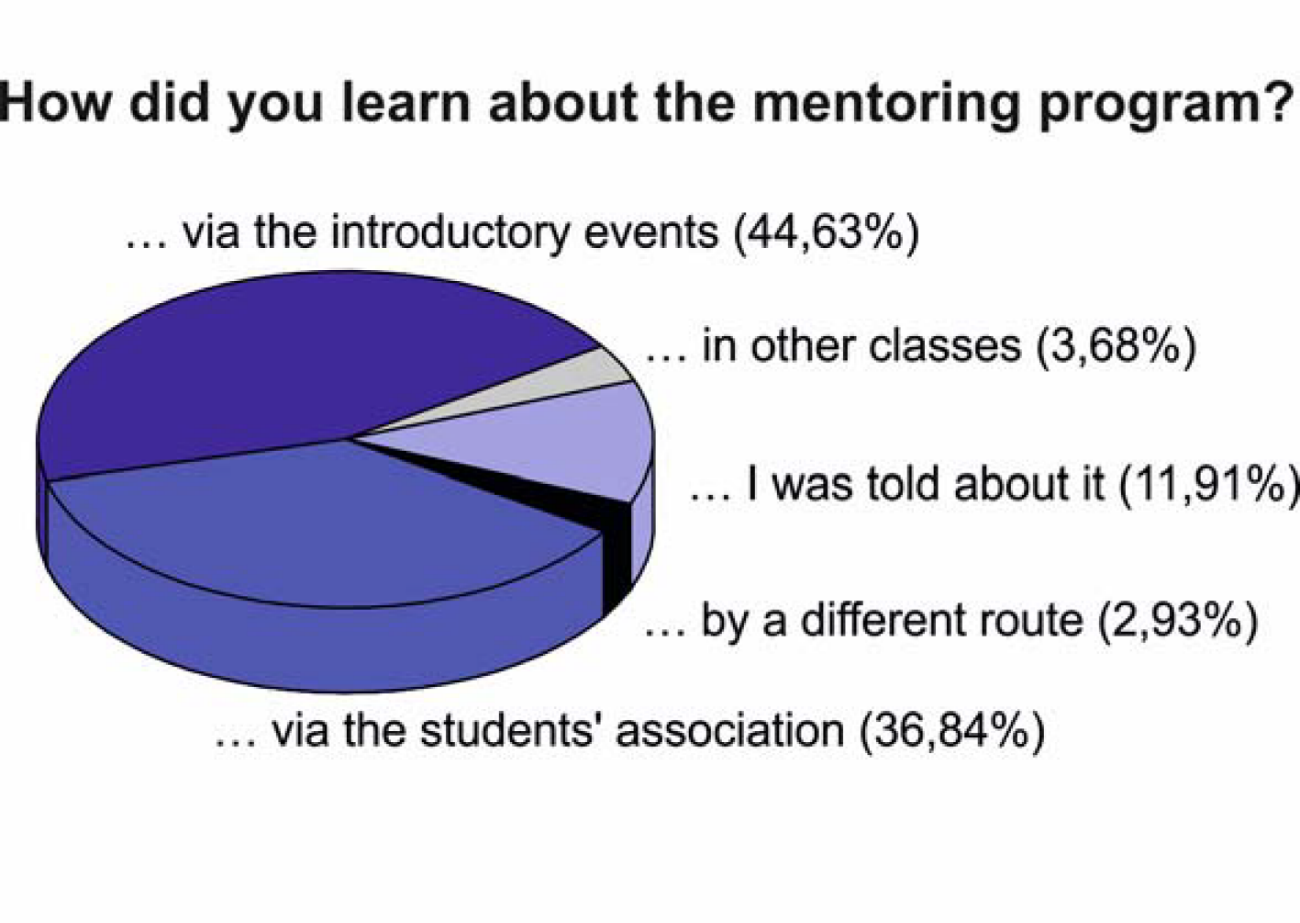
Participants’ source of awareness of the mentoring program across all winter and summer semesters. Multiple answers were possible, data were normalized to 100%.

**Figure 2:**
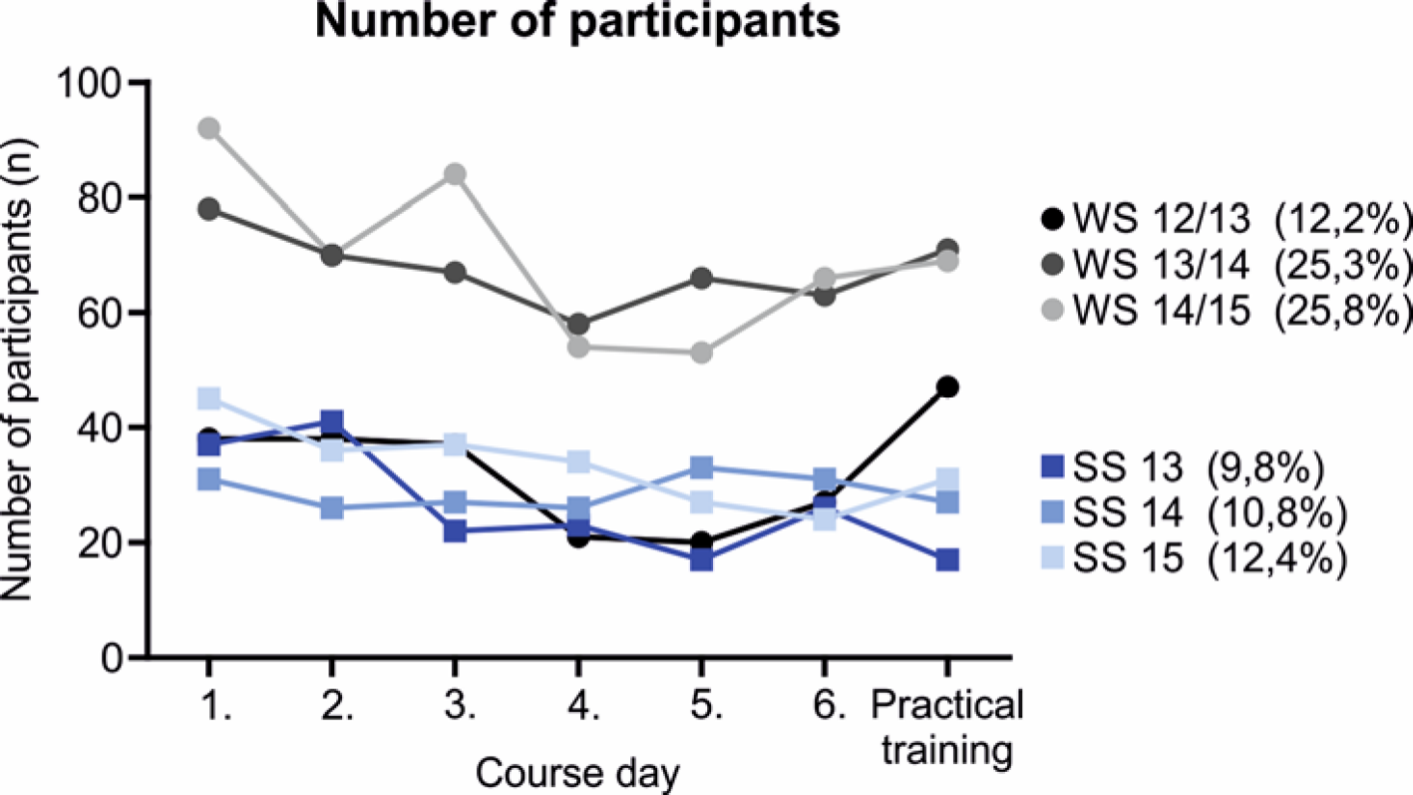
Absolute number of participants over time, across all semesters and the 6 days of the seminar as well as the practical day. Number of participants as percentage of the total number of students in the semester averaged across all semester days are provided in parentheseses.

**Figure 3:**
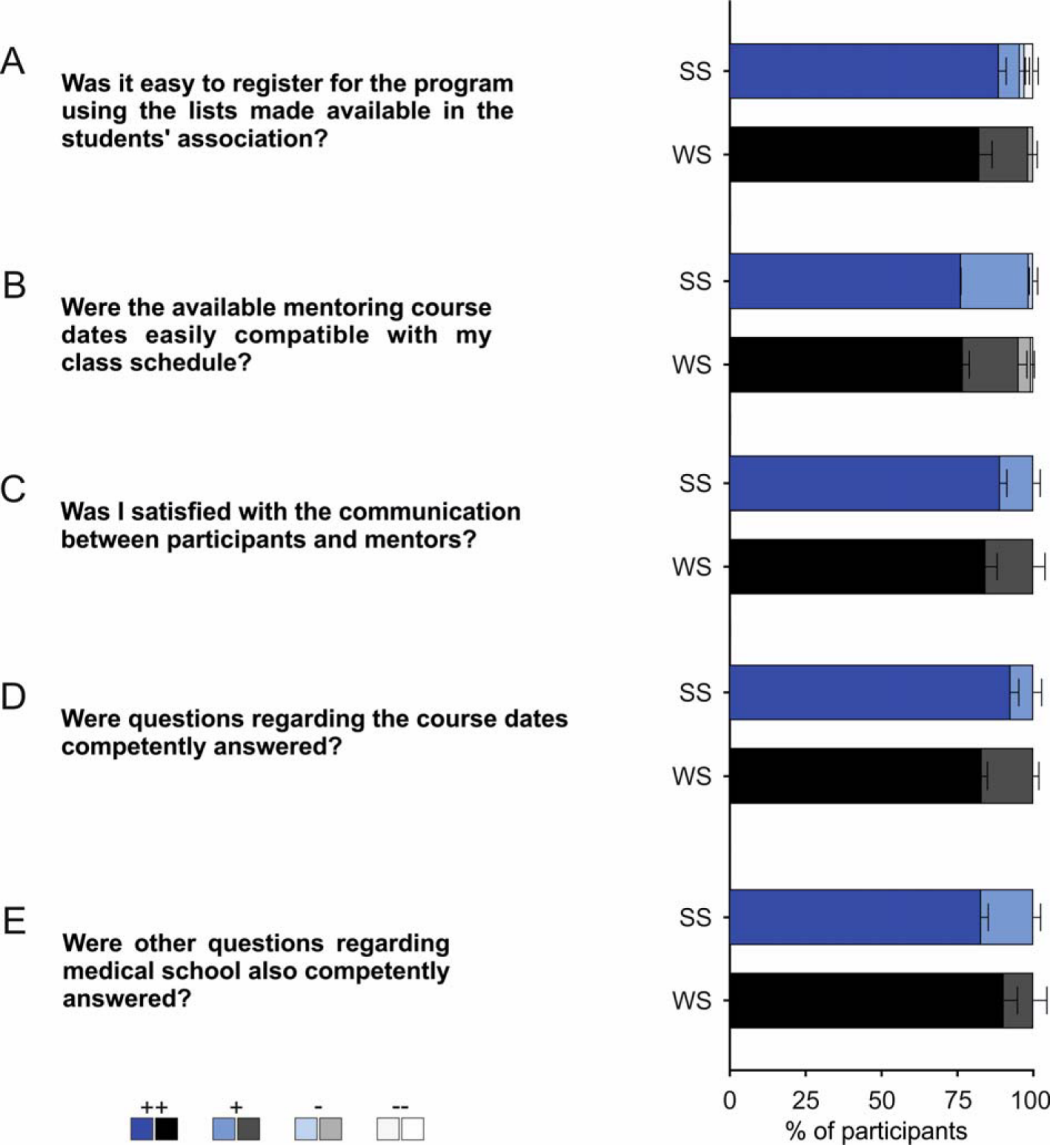
Evaluation of the questionnaires. Results of the evaluation regarding questions for the sign-up procedure (A), for the provided course dates (B), as well as communication between participants and mentors (C). In addition, evaluation of the mentors’ competency in the subject matter (D), and help for general student matters (E). n=3 for winter semester and summer semester, respectively.

**Figure 4:**
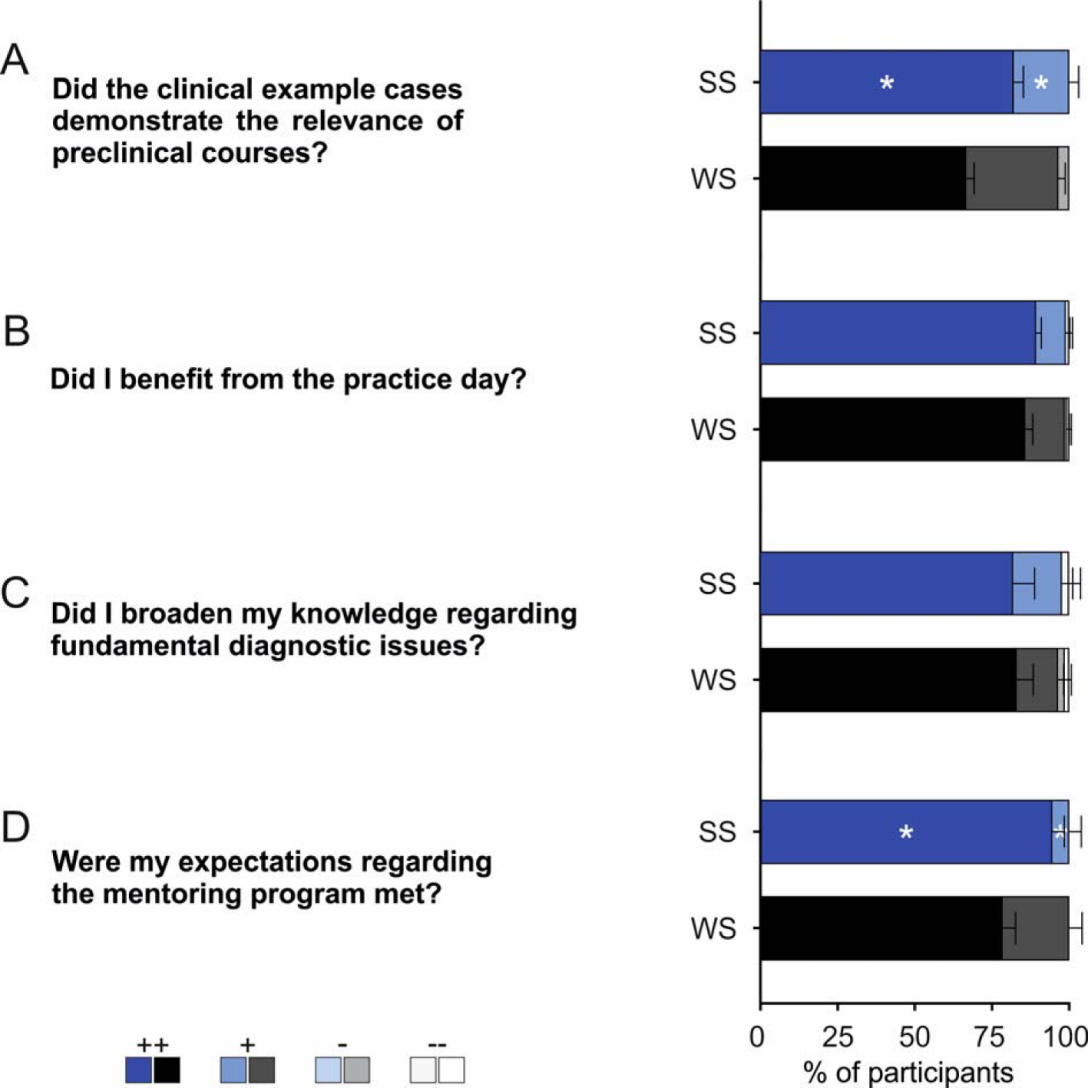
Evaluation of the questionnaires: Did the mentors succeed in demonstrating the relevance of the preclinical subjects (A), evaluation of the practical day and whether students benefit from it (B) and whether they were able to extend their basic diagnostic knowledge (C). (D) shows whether the participants expectations were met. n=3 for winter semester and summer semester, respectively. (*) indicates a significant difference between the summer semester as compared to the winter semester (see results section).

The initial important question to be answered was how did the preclinical students – subsequently referred to as participants – come to know about this optional mentoring program. Interestingly, as summarized across all WS and SS (see Figure 1 (Fig. 1); multiple answers possible; for better presentation data are normalized to 100%), participants learned of the mentoring program primarily from official sources of information, such as the introductory events by the Dean’s office (44.63%) and the students association (36.84%). The total number of participants increased over the years, while during the course of a semester the number slightly decreased. In addition, the optional mentoring program was noticed by more students during the first preclinical semester (WS) than in the second semester (SS; see Figure 2 (Fig. 2); number of participants as percentage of the respective total number of students in the semester provided in parentheses). The enrollment procedure described above was evaluated by more than 80% of participants in the WS and SS, respectively, as very good (see Figure 3 (Fig. 3), Point A: WS: ++82.3±4.2%, +16.0±3.0%, -1.7±1.2%, SS: ++88,7±2.4%, +6.9±1.8%, -1.6±1.6%, --2.7±1.6%; n=3 for WS and SS, respectively). Furthermore, the time slots offered for the seminars could be well incorporated into the participants‘ schedules (see Figure 3 (Fig. 3), Point B: WS: ++76.8±2.1%, +18.3± 2.6%, -4.1±0.5%, --0.8±0.4%, SS: ++76.2±1.4%, +22.3±0.1%, -1.5±1.5%; n=3 for WS and SS, respectively). Communication between mentors and participants was one of the main aspects of the mentoring program stressed from the beginning. This was considered by more than 80% of participants in the WS and SS, respectively, as very good and was never considered bad or very bad (see Figure 3 (Fig. 3), Point C: WS: ++84.2±3.9%, +15.7±3.9%, SS: ++89.0±2.3%, +11.0±2.3%; n=3 for WS and SS, respectively). Besides the general approachability of the mentors with questions and problems, the participants were asked in the questionnaire to evaluate the quality and usefulness of the mentor’s tips/hints. The mentors’ answers were always evaluated as good or very good, both regarding the content of the individual seminar days (see Figure 3 (Fig. 3), Point D: WS: ++83.1±1.8%, +16.9±1.8%, SS: ++92.5±2.8%, +7.5±2.8%; n=3 for SS and WS, respectively) and regarding other questions related to medical school (see Figure 3 (Fig. 3), Point E: WS: ++90.3±4.5%, +9.7±4.5%, SS: ++82.8±2.4%, +17.2±2.4%; n=3 for WS and SS, respectively). Subjects presented on the course days are supposed to make participants directly apply knowledge gained in the preclinical basic sciences to clinical cases and thus to emphasize the relevance of these subjects to the medical profession. This goal was evaluated by participants during the summer semester significantly more positively (as indicated by a higher proportion of “++” and lower “+” evaluations) than during the winter semester (see Figure 4 (Fig. 4), Point A: WS: ++66.7±2.6%, +29.9±2.2%, -3.4±0.4%, SS: ++82.1±3.1% -p=0.02, +17.9±3.1% -p=0.039; n=3 for WS and SS, respectively). Only a small proportion considered this unsuccessful during the winter semester.

**Figure 5:**
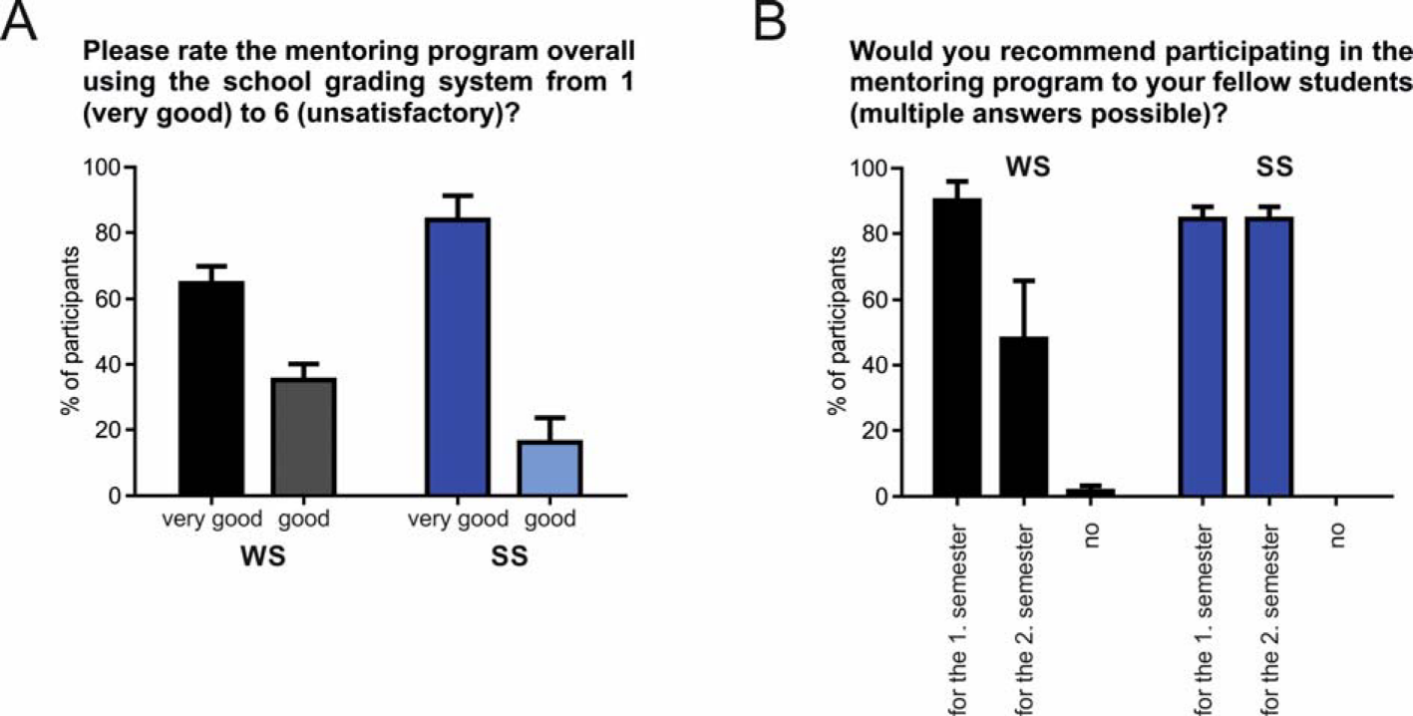
Evaluation of the questionnaires. Overall grade assigned to the mentoring program by participants (A). Percentage of participants who would recommend the mentoring program to fellow students. Multiple responses possible (B). n=3 for winter semester and summer semester, respectively.

After every seminar meeting, participants were asked several questions on the content of the respective seminar. Here, by example, are described the seminar days with the best and worst evaluations, respectively (measured as the proportion of “very good” answers to the question “This subject was very interesting to me.”). Across the winter semesters, the seminar on the subject “colchicine poisoning” received the best evaluation (74.8±4.0%; n=3), whereas the topic “Kidney stones” fared worst (60.5±5.1%; n=3). During the summer semesters, the seminar on “methanol poisoning” received the best results (86.1±0.4%; n=3), the least interesting according to the feedback was on “Spinal disc prolapse“ (53.6±8.5%; n=3). The practical days offered during the winter and summer semesters, in addition to the case-based seminars, were well received. Results showed that participants predominantly benefited from the offered program during both days during the winter and summer semesters (see Figure 4 (Fig. 4), Point B, WS: ++85.8±2.4%, +12.8±1.0%, -0.7±0.7%, --0.7±0.7%, SS: ++89.3±1.7%, +9.6±1.3%, --1.1±1.1%, n=3 for WS and SS, respectively) and were able to extend their knowledge of fundamental / basic diagnostic testing (see Figure 4 (Fig. 4), Point C: WS: ++83.0±5.4%, +13.4±1.8%, -2.1±2.1%, --1.4±1.4%, SS: ++81.9±7.0%, +15.8±6.0%, -- 2.3±1.2%, n=3 for WS and SS, respectively). Finally, participants from every semester were asked to evaluate whether their expectations of the mentoring program, which they generated through different sources of information at the beginning of the semester (see Figure 1 (Fig. 1)), were met by the program and by the mentors. Participants always responded with “good” or “very good” – with a significantly higher proportion of “++” and therefore lower “+” during the summer as compared to the winter semesters (see Figure 4 (Fig. 4), Point D: WS: ++78.5±4.2%, +21.5±4.2%, SS: ++94.6±3.9% -p=0.048, +5.4±3.9% -p=0.048; n=3 for WS and SS, respectively). This result was also reflected when an overall grade was assigned to the mentoring program using the German school grading system (see Figure 5 (Fig. 5), Point A: WS: very good 64.8±5.0%, good 35.2±5.0%, SS: very good 83.9±7.5%, good 16.1±7.5%; n=3 for WS and SS, respectively). For the future of the mentoring program, participants were asked whether they would recommend the program to fellow students (see Figure 5 (Fig. 5), Point B: WS: for first semester 90.2±5.8%, for second semester 48.0±17.9%, no 1.7±1.7%, SS: for first semester 84.4±3.8%, for second semester 84.4±3.8%; n=3 for WS and SS, respectively; multiple answers possible). More than 90% of participants during the first preclinical semester (WS) would recommend participation during the first semester und half also for the upcoming second preclinical semester (SS). Participants during the second preclinical semester (SS) would recommend participation during both semesters.

The test simulation toward the end of the fourth preclinical semester cannot be objectively evaluated currently due to data protection of the actual pass rates of the participants of the simulation and also due to the limited numbers of semesters at this stage (not yet n=3 for WS/SS). Participants were here asked to complete a questionnaire on the quality of the questions, on the created test atmosphere, and to provide a school grade. At the beginning of the next semester all participants were asked with their consent by email, how they would retrospectively assess the test simulation and whether they benefited from it for the actual oral test. Due to missing data, we only provide by way of example the good evaluation as assessed by the school grade system on the questionnaire (67% very good, 33% good during the WS13/14; and 69% very good, 31% good during the WS14/15, no statistics). Further data need to be collected.

## Discussion

The mentoring program was developed as an additional aid for motivation and learning for students during the preclinical years of medical school. For that reason, individual dates for the seminars were offered several times per week so that participants could pick a date compatible with their schedule. Thus, all students had the opportunity to participate in the mentoring program. With this approach, it was possible to increase the number of participants over time. A clear difference was observed between the winter and summer semesters. At Bonn University, the first preclinical semester is during the winter semester, and so there is, besides questions on the seminar-related subjects, an increased demand for exchange with an experienced mentor on general questions regarding medical school and to solve problems. During the second preclinical semester (during the summer semester) there was a corresponding decrease in the number of participants. This is also emphasized by the observation that participants during the first semester predominantly recommend the mentoring program, whereas during the second semester, after familiarization, this is clearly less so. Based upon the fact that the predominant number of participants during the second semester had already participated during the first semester, one can conclude that these participants were primarily concerned with the integrated learning experience and that they correspondingly recommended the seminars in both semesters.

Good cooperation with official bodies of the university were of special importance [12]. Most participants learned about the mentoring program during the official semester introductory events of the deanery and of the students‘ association. Furthermore, cooperation with other institutes led to skilled elaboration and, at times, review of the case examples [13], by which a high degree of expertise could be achieved especially for the mentors’ answering questions. It should be made clear that while the mentors highly valued the exchange of information with the respective basic science departments, a clear separation from the usual curricular teaching was kept. The voluntary seminar meetings were not intended to be tutorials with the aim of better preparing students for the examinations in the individual subjects, but rather were to be an opportunity to apply already acquired basic knowledge to cases as seen in the later professional life. Using a flexible and variable organization/presentation of the seminar meetings, the participants were shown the relevance of the preclinical subjects. Simultaneously, by the preclinical-clinical connection fundamental skills [14], such as taking a structured history and working up a rational differential diagnosis, could be learned and promoted already from the start of medical school. Participants of the mentoring program could thus be given more confidence regarding the clinical work-up and practices as encountered in routine practice. Participants are earlier capable of working on a more independent level with patients during electives and in the practical year at the end of medical school [15]. The subjects and exercises offered on the practice day also contribute to this.

The mentoring program was officially evaluated by the ZEM of Bonn University from its inception. However, due to its voluntary nature, two fundamental systematic errors have to be considered when evaluating the statistical analysis of the quality assurance. A voluntary supplementary offer is subject to an obvious selection bias, and the lack of an evaluation of participants who dropped out limits a fair comparison with compulsory curricular classes. This needs to be considered critically. Nevertheless, analyzing participants’ satisfaction [16] has two important effects. First, mentors use the evaluations as a flexible quality management system, to evaluate and scrutinize continuously their course offerings (replacement of poorly evaluated case examples, improvements of existing cases, selection of subjects, and communication with the students) [17]. Second, the students‘ wishes and ideas can be addressed, such as with the introduction of the “EMMA” seminars. Furthermore, positive as well as negative feedback are presented objectively and can be provided to official councils/boards of the university and the Dean’s office when requesting an extension of the mentors‘ positions and thus a continuation of the program. 

## Conclusion

The concept of peer-to-peer teaching in the context of a preclinical mentoring program at Bonn University has fulfilled students’ expectations. The mentoring program combines the positive characteristics of peer-to-peer teaching [18], which have been illuminated repeatedly in learning and teaching theory [19], with an increase in motivation and learning progress. Besides the „young peer teacher vs. senior expert“, other decisive factors in favor of the program were “same level” education, and a practical deepening of the study subjects in combination with a pleasant atmosphere for learning and the transfer of experiences. 

In summary, since its introduction the mentoring programing has been very well received by the preclinical students and enriches the Bonn medical school curriculum in a multifaceted way. 

## Authors

All the authors contributed equally to the manuscript.

## Acknowledgements

For the realization of this project we sincerely thank the former preclinical coordinator Dr. rer. nat. C. Stieber as well as the current coordinator Dr. rer. nat. M. Breitbach. Furthermore, we thank Prof. Dr. D. Swandulla (Institute for Physiology 2, Bonn University) for the employment and support of the mentors as well as Prof. Dr. V. Gieselmann for his support in his former role as Prorector (Studium und Lehre). A special Thank you to the former and active mentors who with their commitment continuously developed and improved this project (Sandra Breidenich, Katharina Bullok, Sabrina Drouven, Alexandra Herbster, Vici Hess, Jörgen Hoffmann, Julian Hußmann, Lukas Kunz, Tatjana Lauck, Maren Lieberz, Anna Lohmann, Carolin Loth, Hannah Lucht, Anna Metzner, Pia Nordmann, Laurèl Rauschenbach, Mandy Schlauer, Martin Schröder, Judith Schultewolter, Daniel Schumacher, Leon Strauß, Marius Vach, Paul Wesselmann, Maria Willis). Special thanks for the english translation go to Lisa Costello-Boerrigter and Guido Boerrigter.

## Competing interests

The authors declare that they have no competing interests. 

## Figures and Tables

**Table 1:**
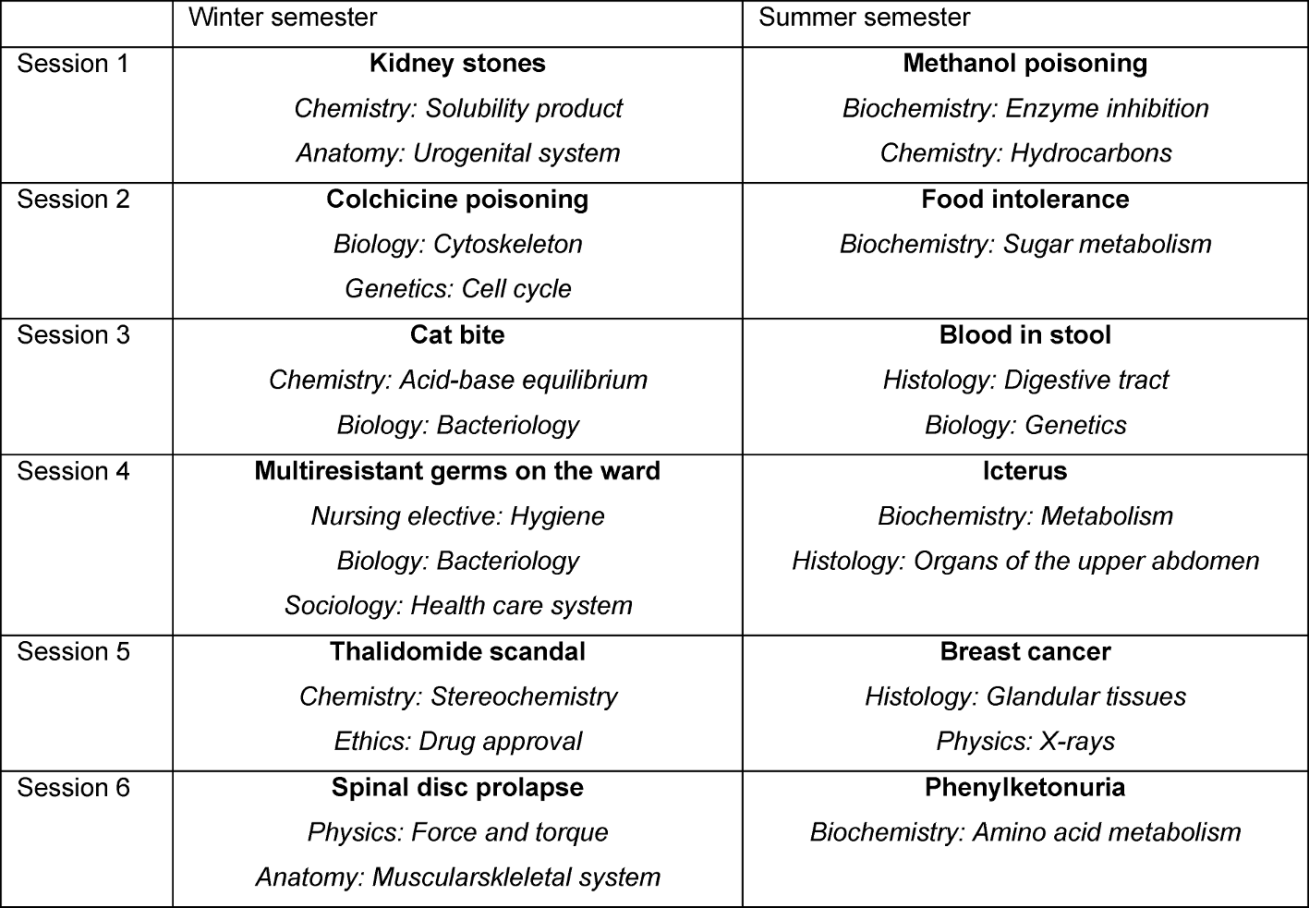
Meeting subjects in the first and second semester (status as of summer semester 2016)
